# miRNA-21 promotes osteogenesis via the PTEN/PI3K/Akt/HIF-1α pathway and enhances bone regeneration in critical size defects

**DOI:** 10.1186/s13287-019-1168-2

**Published:** 2019-02-22

**Authors:** Chi Yang, Xiaohan Liu, Kai Zhao, Youming Zhu, Bin Hu, Yong Zhou, Mohan Wang, Yiqun Wu, Chengfei Zhang, Jianguang Xu, Yujie Ning, Duohong Zou

**Affiliations:** 1 Department of Oral Surgery, Shanghai Key Laboratory of Stomatology, National Clinical Research Center of Stomatology, Ninth People’s Hospital, Shanghai Jiao Tong University School of Medicine, Shanghai, 200011 People’s Republic of China; 2 Second Dental Clinic, Department of Oral Implantology, School of Medicine, Shanghai Key Laboratory of Stomatology, National Clinical Research Center of Stomatology. Ninth People’s Hospital, Shanghai Jiao Tong University, Shanghai, 200011 People’s Republic of China; 3 Department of Dental Implant Center, Stomatologic Hospital & College, Anhui Medical University, Key Laboratory of Oral Diseases Research of Anhui Province, Anhui Province, Hefei, 230032, People’s Republic of China; 4 Endodontology, Faculty of Dentistry, University of Hong Kong, Hong Kong, People’s Republic of China

**Keywords:** miRNA-21, BMSCs, PTEN/PI3K/Akt, Bone regeneration, Bone defects

## Abstract

**Background:**

Functional reconstruction of maxillofacial bone defects is a considerable clinical challenge. Many studies have emphasized the osteogenic and angiopoietic abilities of stem cells for tissue regeneration. We previously showed that microRNA-21 (miRNA-21) can promote angiogenesis in human umbilical cord blood-derived mesenchymal stem cells (UCBMSCs). In the present study, the role of miRNA-21 in osteogenic differentiation of bone marrow-derived stem cells (BMSCs) was investigated.

**Methods:**

Western blotting and qPCR were performed to investigate the influences of miRNA-21 on osteogenic differentiation of BMSCs. The effects of miRNA-21 on PTEN/PI3K/Akt/HIF-1α pathway were also assessed using western blotting. To further evaluate the roles of miRNA-21 in osteogenesis in vivo, we conducted animal experiments in rat and canine. New bone formation was assessed using micro-CT and histological methods.

**Results:**

In the present study, we found that miRNA-21 promotes the migration and osteogenic differentiation of bone marrow-derived stem cells (BMSCs) in vitro. Using gain- and loss-of-function studies, we found that miRNA-21 promoted the osteogenic ability of BMSCs by increasing P-Akt and HIF-1α activation. Finally, we verified the essential role of miRNA-21 in osteogenesis by implanting a miRNA-21-modified BMSCs/β-tricalcium phosphate (β-TCP) composite into critical size defects. Radiography, micro-CT, and histology revealed significantly greater volume of new bone formation in the miRNA-21 group than in the control group.

**Conclusion:**

In conclusion, our study demonstrated an essential role of miRNA-21 in promoting maxillofacial bone regeneration via the PTEN/PI3K/Akt/HIF-1α pathway.

**Electronic supplementary material:**

The online version of this article (10.1186/s13287-019-1168-2) contains supplementary material, which is available to authorized users.

## Background

The maxillofacial bone is an important anatomical component of craniofacial function and morphology [1]. Various factors including congenital malformation, trauma, and tumors can lead to maxillofacial bone defects. In such cases, functional maxillofacial bone reconstruction is complex and difficult. At present, autogenous bone grafts, allografts, and xenografts are considered the most common treatments [2, 3]. However, many complications including donor morbidity and availability, pain, infection, immune rejection, and loss of function limit the clinical application of these techniques [4]. In recent years, tissue engineering has gradually become one of the promising alternatives to traditional bone regeneration techniques.

Genetically modified stem cells have been shown to play significant roles in promoting bone regeneration [5]. Previous studies have reported that a variety of factors, such as transcription factors, growth factors, cytokines, and extracellular matrix molecules, can promote osteogenesis and angiogenesis in bone tissue engineering [6]. miRNAs have recently been reported to participate in many important physiological and pathological processes by regulating complex gene activities [7–10]. Specifically, several miRNAs have been shown to be important for various aging-related diseases, cell differentiation, and bone regeneration [11–13]. Moreover, we previously found that miRNA-21 promoted angiogenesis in human umbilical cord blood-derived mesenchymal stem cells (UCBMSCs) by enhancing hypoxia-inducible factor-1α (HIF-1α) activity [14]. Previous studies largely focused on the functions of miRNA-21 in cardiovascular system formation; the function of miRNA-21 in bone regeneration remains largely unknown.

In the present study, we provide evidence that miRNA-21 can promote osteogenesis in bone marrow-derived stem cells (BMSCs) in vitro. miRNA-21 promoted BMSC osteogenesis via the PTEN/PI3K/Akt pathway. Moreover, we demonstrated that β-tricalcium phosphate (β-TCP) scaffolds seeded with miRNA-21-modified BMSCs improved new bone formation in critical size defects (CSD) in vivo.

## Materials and methods

### Cell culture and Lenti-miR-21(LacZ)-Luciferase construction

BMSCs were obtained from Labrador dogs (approximately 2 years old) using a previously described method and then cultured in DMEM (Sigma-Aldrich, St. Louis, USA) supplemented with 10% fetal bovine serum (FBS), 1% penicillin/streptomycin, and 1 mM l-glutamine (Invitrogen, USA) at 37 °C in a humidified, 5% CO_2_ atmosphere [15, 16]. Lenti-miR-21(LacZ)-Luciferase was constructed as described previously [14]. Cells were plated in 12-well plates (5 × 10^4^ cells/well) prior to infection with Lenti-miR-21(LacZ)-Luciferase or transfection with miR-21 mimics or miR-21 inhibitor [14, 17]. miR-21 mimics (1.6 μg) or inhibitor − 21 (1.6 μg) (Shanghai GenePharma, China) was transfected into BMSCs using Oligofectamine (Invitrogen, USA) according to the manufacturer’s instructions. Cells were collected 48 h after transfection, and the expression of the related proteins was detected.

#### Real-time PCR

For reverse transcription PCR, total RNA was extracted using TRIzol reagent (Sigma-Aldrich, USA). cDNA was synthesized using a PrimeScript RT kit following the manufacturer’s instructions (Takara, Japan). qPCR was performed using SYBR Premix Ex Taq (Takara), and the results were analyzed using a Stratagene Mx3000p system (Agilent Technologies, USA). The miRNA-21 primers and qPCR primer sequences are listed in Additional file 1.

#### Western blotting

Cells were harvested and then heated at 95 °C in a sample loading buffer for 10 min. Proteins were separated via 12% SDS-PAGE, transferred to PVDF membranes (Millipore, USA), and then incubated with fat-free milk. The PVDF membranes were then subjected to immunoblotting using the indicated antibodies. Proteins were visualized using an enhanced chemiluminescence method. The following primary antibodies were used: OPN (ab104302), HIF-1α (ab12289), VEGF (ab46154), Runx2 (ab76596), BMP-2 (ab14933), OCN (ab13420), Akt (Cell Signaling 9272), P-Akt (Signaling 4060), and GAPDH (sc-137179).

#### Rat skull bone formation induced by Lenti-miRNA-21/β-TCP/BMSC scaffolds

All animal experiments were approved by the Independent Ethics Committee of Shanghai Ninth People’s Hospital and Anhui Medical University. All experiments were conducted in accordance with the Division of Laboratory Animal Medicine guidelines. A calvarial bone-defect rat model was created following a previously described method [18, 19]. Briefly, animals were anesthetized via inhalation of 2% isoflurane, and then a linear incision was made along the midline of the skull. Full-thickness flaps were raised, and 5-mm craniotomy defects were created on each side using a trephine. Rat BMSCs were isolated and cultured as described previously [20, 21]. β-TCP scaffolds (National Engineering Research Center of Organizational Engineering, Shanghai, China) with an average pore size of 400 μm ± 50 μm and 75% porosity were used as in our previous study [22]. Rat BMSCs transfected with the lentivirus construct (miRNA-21/ LacZ) were resuspended at a density of 1.0 × 10^7^ cells/mL, seeded into β-TCP scaffolds (200 μL per scaffold), and then immediately transplanted into the defect area of the rat calvarial bone. Bone defects transplanted with β-TCP scaffolds alone or β-TCP scaffolds with BMSCs were used as controls. At 60 days postsurgery, the animals were sacrificed with an overdose of pentobarbital. Specimens were harvested and immediately fixed in a 10% formalin solution. Samples were taken for micro-CT analysis using an animal micro-CT scanner (eXplore Locus, GE Healthcare Biosciences, London, UK) as previously reported [23]. After micro-CT scanning, bone visualizations were reconstructed using three-dimensional (3D) isosurface rendering software. Bone mineral densities (BMDs) and the trabecular thickness (TbTh) were calculated for each group. Then, samples from each group (*n* = 6, total 18) were decalcified in 10% EDTA for 2 weeks. Samples were embedded in paraffin, and serial sagittal sections were made. Three sections were obtained from each sample. After hematoxylin-eosin staining, new bone regeneration was histologically examined using optical microscopy, as previously described [23].

#### Bone regeneration induced by Lenti-miRNA-21/β-TCP/BMSC scaffolds in a canine mandibular defect model

All canine experiments were approved by the Independent Ethics Committee of Shanghai Ninth People’s Hospital and Anhui Medical University and conducted in accordance with the Division of Laboratory Animal Medicine guidelines. Canines were randomly divided into four groups, with three canines per group, anesthetized via intramuscular injection of ketamine (10 mg/kg) and placed in a supine position. The body of the mandible was exposed through a submental midline skin incision. After adjustment of the titanium plate, an osteoperiosteal segmental defect with a size of 20 mm × 10 mm was made at the midportion of the mandible using an oscillating bone saw, as previously described [22]. The neurovascular bundle was cut and stanched by filling the mandibular canal with bone wax. Then, the titanium plate was fixed with two screws (one on the mesial side and one on the distal side). The defects were filled with β-TCP scaffolds, Lenti-LacZ/β-TCP/BMSC scaffold constructs, or Lenti-miRNA-21/β-TCP/BMSC constructs. Empty bony defects served as controls. The incisions were closed with 4–0 silk sutures (Additional file 1: Figure S3). All animals were injected with penicillin for 7 days postsurgery and received a soft diet during the study.

#### Sequential fluorescence labeling

Sequential fluorescence labeling of mineralized tissues was performed as described previously [24–26]. At 3, 6, and 9 weeks postsurgery, fluorochromes were intraperitoneally injected into the 12 canines under anesthesia as follows: 25 mg/kg tetracycline hydrochloride (Amresco, USA), 20 mg/kg calcein (Amresco, USA), and 30 mg/kg Alizarin Red S (ARS, Sigma-Aldrich, USA).

#### Radiographic observations

Mandibular X-ray images of experimental animals were obtained at 2 weeks, 3 months, and 6 months postsurgery to assess scaffold degradation, new bone formation, and mineralization of the defect area as previously described [23]. Radiographs were obtained using a dental X-ray machine (Trophy, France) at 7 cm (230 V, 8 mA), with an exposure time of 0.28 s.

#### Micro-CT analysis

Six months postsurgery, the 12 canines were euthanized with excessive anesthesia; mandibular specimens were then obtained and were immediately fixed in 10% formalin. The samples were scanned using a micro-CT device and reconstructed into 3D images to display gross morphology, as previously described [23]. The samples were scanned using a micro-CT system (GE eXplore Locus SP Micro-CT, USA). Scanning parameters were set at 80 kV and 80 μA, with an exposure time of 3000 ms and a pixel size of 15 μm. After scanning, 3D images were reconstructed using GEHC MicroView software (GE Healthcare Biosciences, Chalfont St. Giles, UK). To quantify the new bone formed around the scaffolds, a 20 mm × 10 mm region was analyzed for BMD, bone volume fraction (bone volume/total volume, BV/TV), TbTh, and trabecular number (TbN).

#### Histomorphometric observations

After micro-CT scanning, the undecalcified mandibular specimens were dehydrated using graded alcohol solutions (75% to 100%) and finally embedded in PMMA. Sections were cut along the sagittal surface in each sample. Three sections, representing the central area of each defect, were used for histometric analysis. The embedded specimens were cut into 150-μm-thick sections using a Leica SP1600 microtome (Leica, Hamburg, Germany). The sections were subsequently polished to a final thickness of approximately 40 μm and observed via confocal laser scanning microscopy (CLSM, Leica TCS Sp2 AOBS, Heidelberg, Germany) [23, 27]. The excitation/emission wavelengths were 405/560e590 nm (tetracycline, yellow), 488/500e550 nm (calcein, green), and 543/580e670 nm (ARS, red). Undecalcified sections were stained using van Gieson’s picrofuchsin [23]. Image Pro 5.0 (Media Cybernetic, Silver Springs, MD, USA) was used to measure the areas of newly formed bone and remnant scaffold, and then the percentage of the whole bone-defect area was reported.

#### Statistical analysis

All experiments were performed in triplicate, unless otherwise specified. The results are presented as the means ± standard deviation (SD). Statistical significance was assessed by analysis of variance (ANOVA) with Tukey’s post hoc test, and *p* < 0.05 was considered statistically significant (*p < 0.05 and ^#^*p* < 0.05; experimental groups compared with the control group).

## Results

### Expression of osteogenic- and angiogenic-related genes in miRNA-21-modified BMSCs

**Fig. 1:**
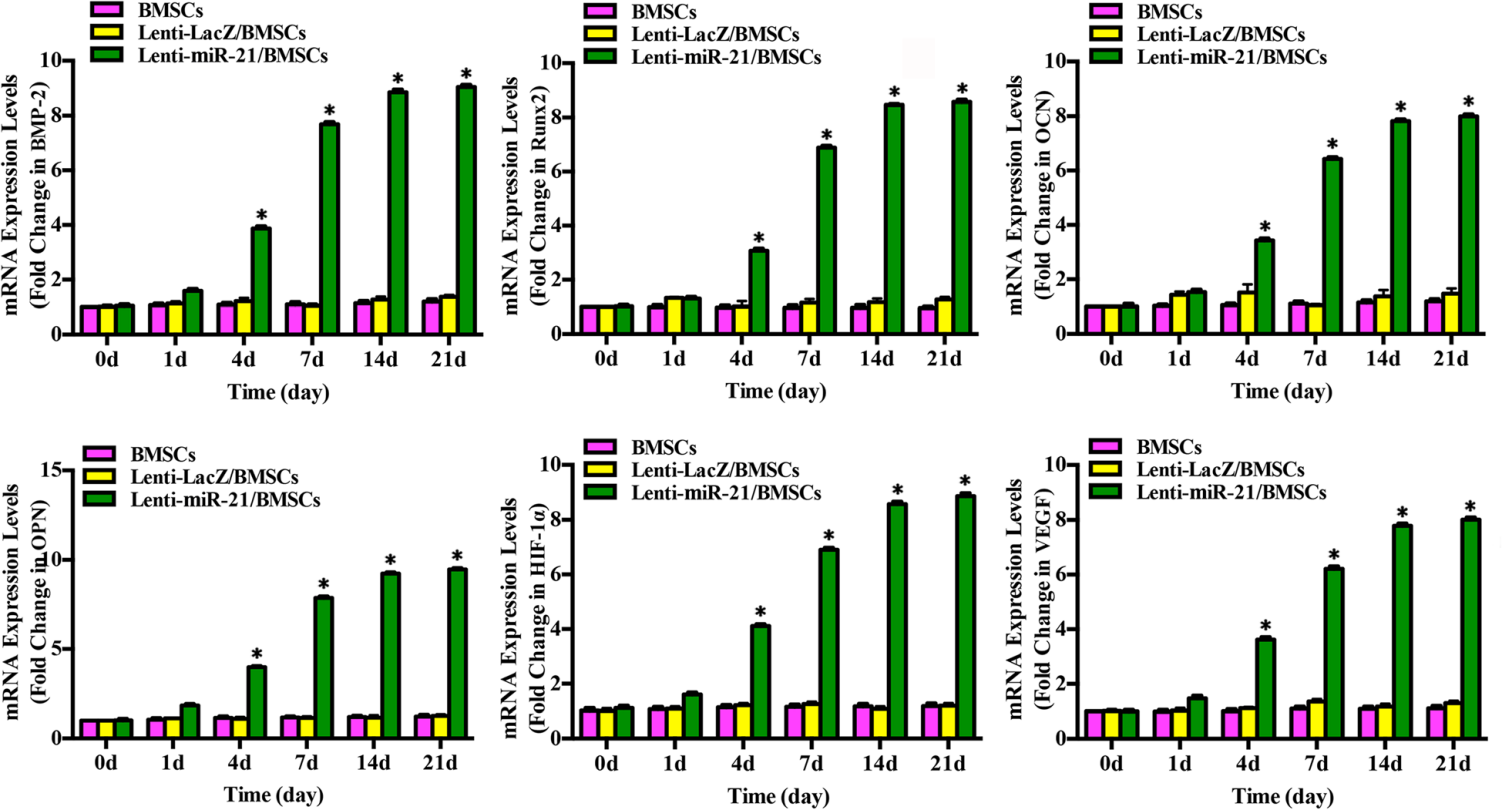
Expression of osteogenic mRNAs in BMSCs in vitro. After BMSCs were infected with Lenti-LacZ or Lenti-miRNA-21 at the indicated time points, BMP-2, Runx2, OCN, OPN, HIF-1α, and VEGF mRNA expression levels were detected by qRT-PCR

To assess the expression levels of osteogenic mRNAs, BMSCs were infected with Lenti-miRNA-21, and qPCR was performed at 0, 1, 4, 7, 14, and 21 days after transfection (Fig. 1). We found that BMP-2, Runx2, OCN, and OPN mRNA levels increased slightly by day 4 and peaked by days 14 and 21. To further determine whether miRNA-21 can regulate BMSC angiogenic differentiation by regulating HIF-1α and VEGF expression, HIF-1α and VEGF mRNA expression was analyzed. It was revealed that HIF-1α and VEGF mRNA levels were upregulated at 4 days after Lenti-miRNA-21 infection. Compared with those of the Lenti-LacZ group, the levels of these mRNAs were notably increased from day 7 to day 21 in the Lenti-miRNA-21 group.

### Osteogenic differentiation of miRNA-21-modified BMSCs

**Fig. 2:**
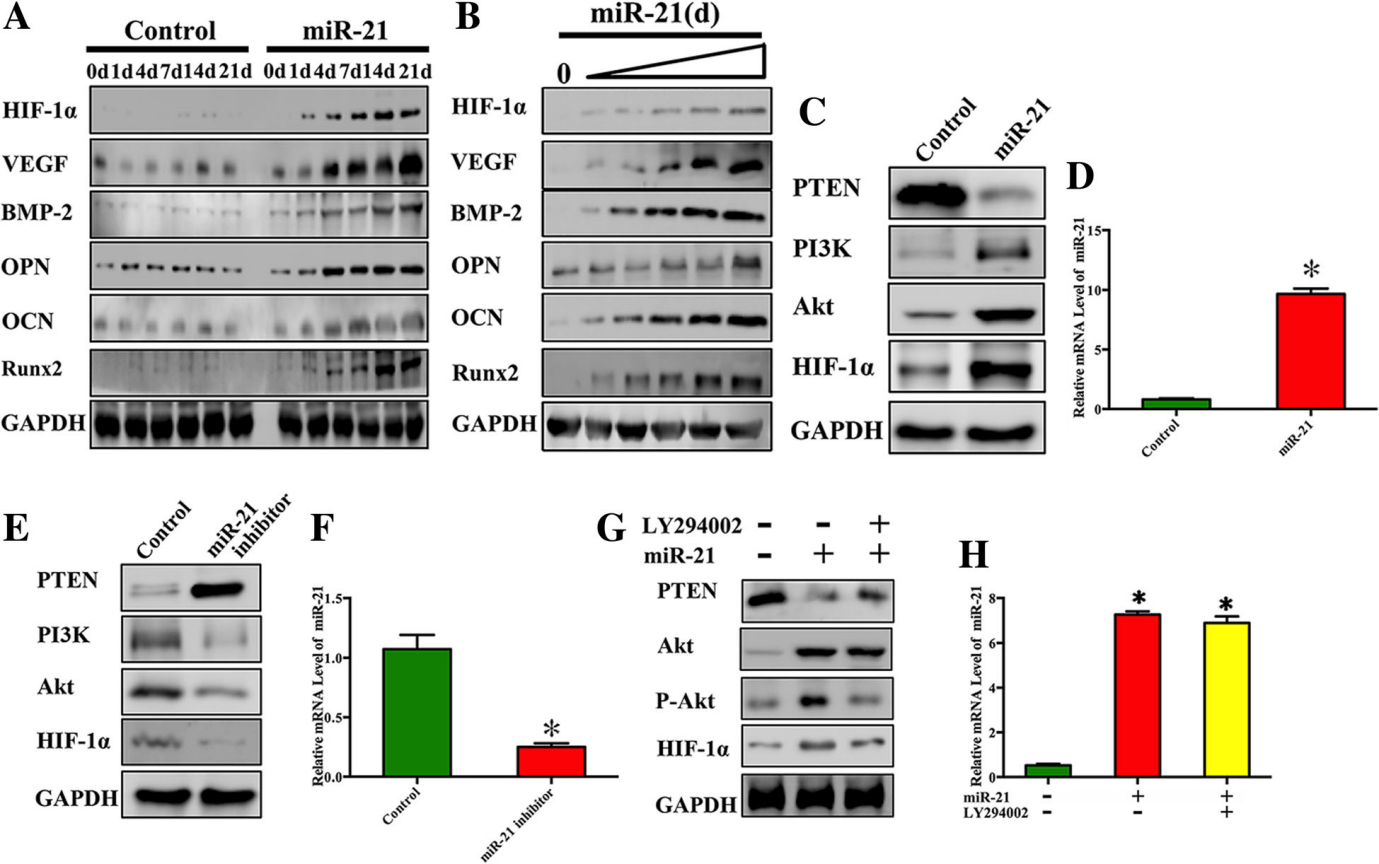
miRNA-21 promotes osteogenesis by upregulating P-Akt expression levels. **a** HIF-1α, VEGF, BMP-2, OPN, OCN, and Runx2 protein levels were detected via western blotting after BMSCs were infected with Lenti-LacZ or Lenti-miRNA-21 at the indicated time points. GAPDH served as the loading control. **b** UCBMSCs were transfected with 0 μg, 0.2 μg, 0.4 μg, 0.8 μg, 1.6 μg [4], or 3.2 μg of miR-21 mimics at 0 day and 7 days, and cells then were collected at 14 days. HIF-1α, VEGF, BMP-2, OPN, OCN, and Runx2 protein levels were detected via western blotting. GAPDH served as the loading control. **c** PTEN, PI3K, Akt, and HIF-1α protein levels were detected via western blotting at 48 h after BMSCs were transfected with control or miRNA-21 mimics. GAPDH served as the loading control. **d** Histogram showing the relative miRNA-21 levels detected by qRT-PCR after BMSCs were transfected with a control or miRNA-21 mimics. **e** PTEN, PI3K, Akt, and HIF-1α protein levels were detected via western blotting at 48 h after BMSCs were transfected with control or miRNA-21 inhibitor. GAPDH served as the loading control. **f** Histogram showing the relative miRNA-21 levels detected by qRT-PCR after BMSCs were transfected with a control or miRNA-21 inhibitor. **g** PTEN, P-Akt, Akt, and HIF-1α protein levels were detected via western blotting after BMSCs were transfected with control or miRNA-21 mimics or exposed to LY294002. GAPDH served as the loading control. **h** Histogram showing the relative miRNA-21 levels detected by qRT-PCR after BMSCs were transfected with control or miRNA-21 mimics or treated with LY294002

To verify that miRNA-21 promotes osteogenic protein expression, western blotting was conducted at 0, 1, 4, 7, 14, and 21 days (Fig. 2a). BMP-2, Runx2, OCN, OPN, HIF-1α, and VEGF expression was increased nearly twofold from day 4 to day 21 in the miRNA-21 group compared with that in the control group.

To evaluate whether miR-21-induced osteogenic protein expression was dose-dependent, we transfected UCBMSCs with various doses of miR-21 mimics. The results showed that the expression of osteogenic proteins was elevated with the dose increment of miR-21. This result suggested that miR-21 overexpression is associated with the upregulation of osteogenesis-related proteins (Fig. 2b).

To investigate the relationship between miRNA-21 and the PTEN/PI3K/Akt pathway, gain- and loss-of-function studies were performed. BMSCs were transfected with miRNA-21 mimics or miRNA-21 inhibitor. miRNA-21 levels in the induced BMSCs increased 10-fold after transfection with the miRNA-21 mimics and decreased nearly threefold after transfection with the miRNA-21 inhibitor. At the same time, western blotting revealed that HIF-1α, Akt, and PI3K protein levels increased nearly twofold after miRNA-21 mimic transfection. Meanwhile, the expression levels of PTEN, an inhibitor of PI3K, decreased after miRNA-21 mimic transfection. In contrast, when BMSCs were transfected with the miRNA-21 inhibitor, these protein levels were downregulated (Fig. 2c–f).

To further validate the relationship between miRNA-21 and PTEN/PI3K/Akt pathway, BMSCs were transfected with miRNA-21 mimics with or without treatment with LY294002 (PI3K inhibitor). miRNA-21 levels in the induced BMSCs increased eightfold after miRNA-21 mimic transfection and showed a similar increase when treated with PI3K inhibitor. At the same time, western blotting revealed that HIF-1α and P-Akt protein levels increased after miRNA-21 mimic transfection and decreased markedly in the presence of PI3K inhibitor. These findings show the reciprocal association between miRNA-21 and the PTEN/PI3K/Akt/HIF-1α pathway (Fig. 2g, h).

### miRNA-21-seeded scaffolds repaired calvarial bone defects in rats

**Fig. 3:**
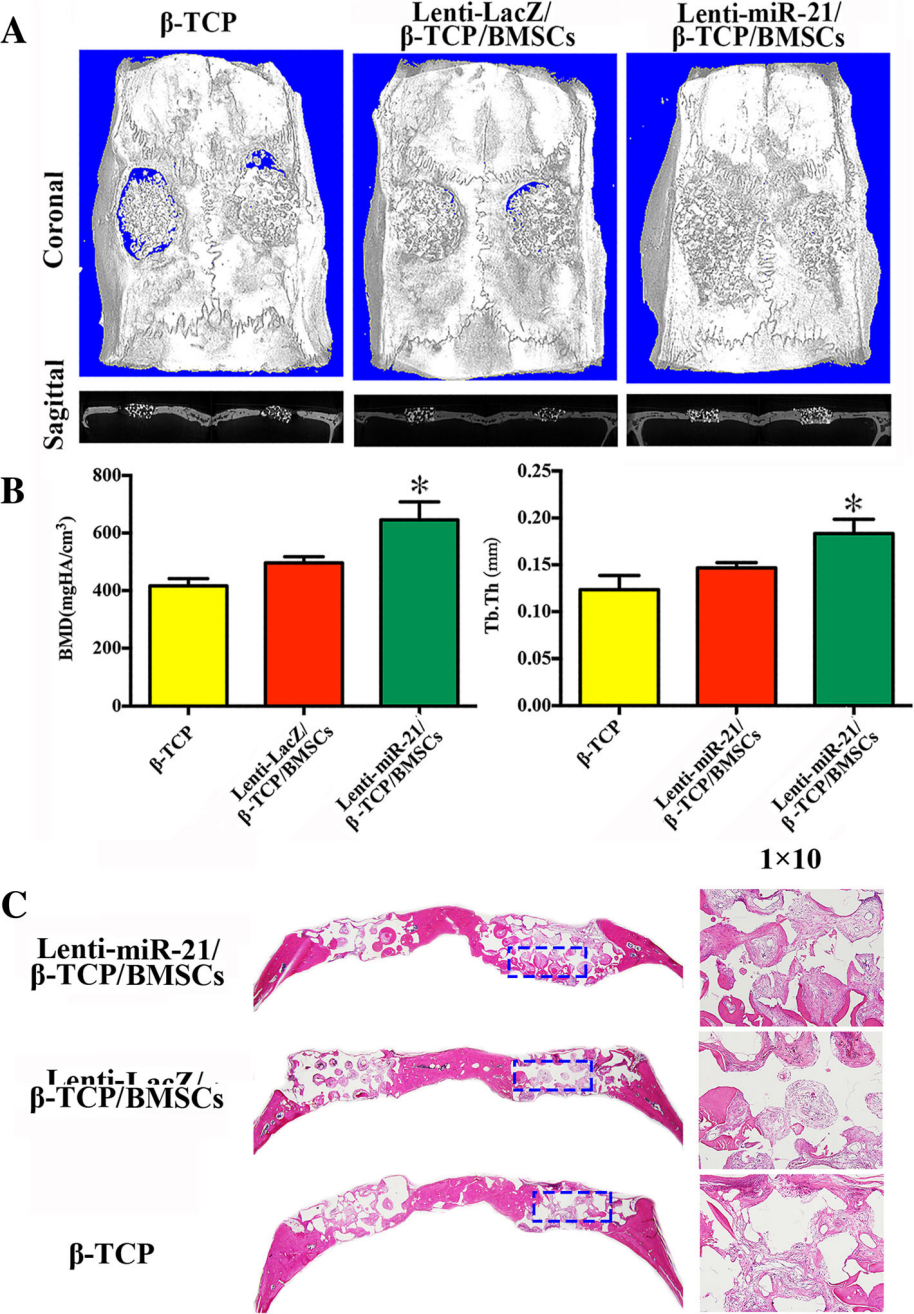
miRNA-21-seeded scaffolds repair calvarial bone defects in rats. **a** Micro-CT images of newly formed bone in the Lenti-miRNA-21/β-TCP/BMSC, Lenti-LacZ/β-TCP/BMSC, and β-TCP groups. **b** Quantitative analyses of the BMD and TbTh of the newly formed bone in the three groups (six animals in each group). The experiment was repeated three times. **c** Histomorphological observations of the newly formed bone in the three groups

To investigate whether miRNA-21 can promote bone regeneration in vivo, Lenti-miRNA-21/β-TCP/BMSC scaffolds were implanted into rat calvarial bone defect sites. By day 60, micro-CT observations (Fig. 3a, b) revealed newly formed bone in the Lenti-miRNA-21/β-TCP/BMSC and Lenti-LacZ/β-TCP/BMSC scaffold groups. Furthermore, new bone formation in the calvarial bone defect area was analyzed via histological examination (Fig. 3c), which revealed that much more new bone formed in the Lenti-miRNA-21/β-TCP/BMSC scaffold implantation group compared with the other two scaffolds. Similarly, micro-CT images revealed more new calvarial bone formation in the Lenti-miRNA-21/β-TCP/BMSC scaffold group. Furthermore, quantitative analyses demonstrated that BMD and TbTh were significantly higher in the Lenti-miRNA-21/β-TCP/BMSC group compared with the Lenti-LacZ/β-TCP/BMSC group, indicating that miRNA-21 promotes osteogenesis in BMSCs.

### Radiographic and micro-CT observation of miRNA-21-induced bone regeneration in canine mandibular defects

To assess the effect of miRNA-21 on bone regeneration, three groups of combinations (β-TCP, β-TCP with Lenti-LacZ/ BMSCs, and β-TCP with Lenti-miRNA-21/BMSCs) were placed into canine alveolar bone defects.

**Fig. 4:**
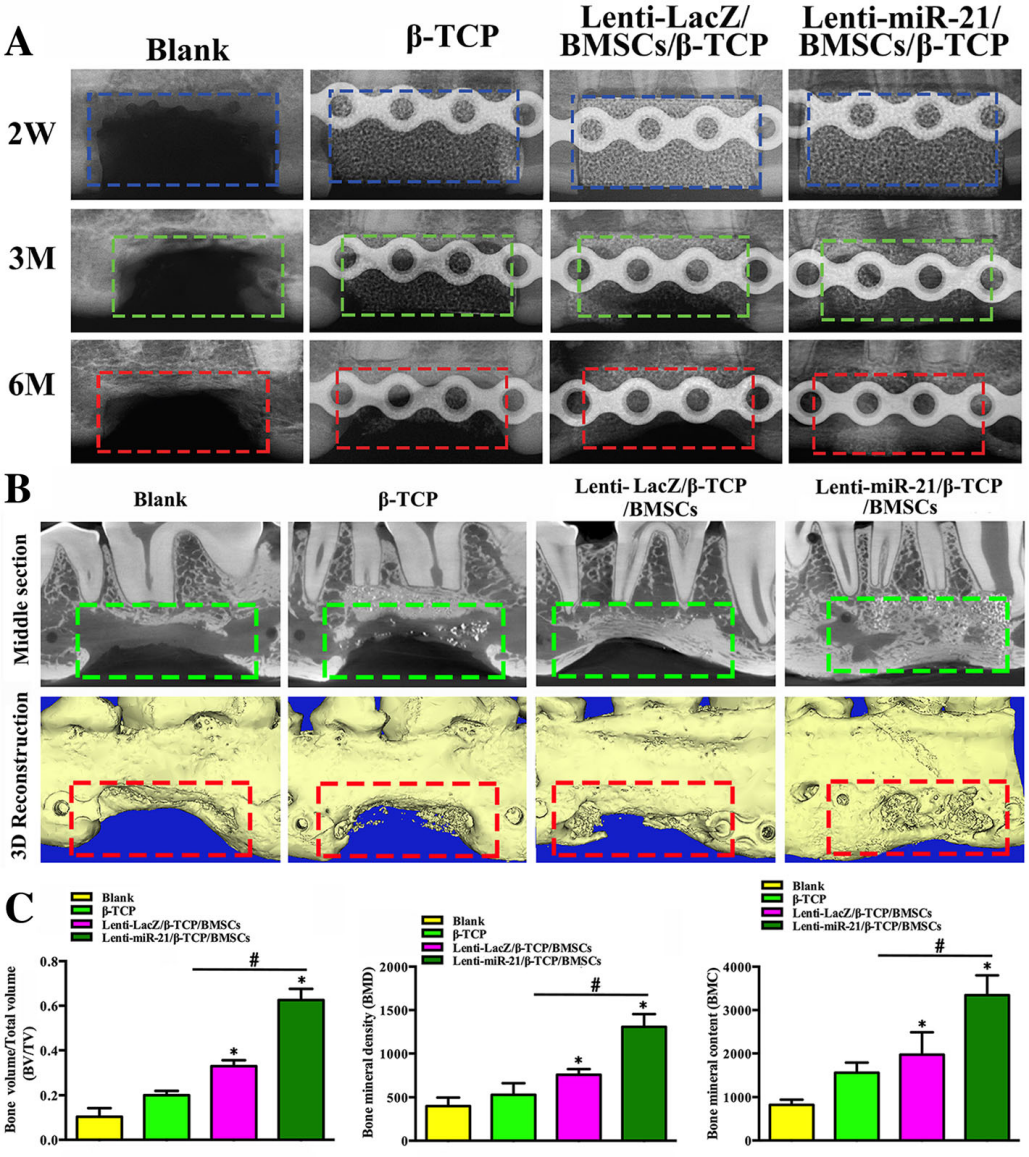
Radiographic and micro-CT observation and analysis of miRNA-21-induced bone regeneration in canine mandible defects. **a** Radiographic observations of β-TCP, Lenti-LacZ/β-TCP/BMSC, and Lenti-miRNA-21/β-TCP/BMSC scaffolds 2 weeks, 3 months, and 6 months postoperation. A sham operation group served as the control. **b** Cross-section micro-CT images of canine mandibles with scaffolds 6 months postoperation. **c** Quantitative results from micro-CT evaluations of the four groups (three animals in each group). The experiment was repeated three times. The green dotted pane denotes micro-CT images of the middle sections of canine mandibular defects. The red dotted pane indicates 3D reconstruction of canine mandibular defects (*p < 0.05; ^#^*p* < 0.05)

New bone formation in the β-TCP, Lenti-LacZ/β-TCP/BMSC, and Lenti-miRNA-21/ β-TCP/BMSC groups was evaluated radiographically at 2 weeks, 3 months, and 6 months postoperation. A sham operation group served as the control (Fig. 4a). New bone formation was greater in the miRNA-21 group than in the other groups.

To observe new bone formation within the defects, canine mandibular bones were scanned using micro-CT 6 months postoperation (Fig. 4b). In the Lenti-miRNA-21 group, the canine mandibular defects were filled with newly formed bone, and the implants were also surrounded with new bone. However, different gap sizes were observed in the peripheral areas of the implants for the sham, β-TCP, Lenti-LacZ, and Lenti-miRNA-21 groups. From the middle view, greater new bone formation was observed in the Lenti-miRNA-21 group than in the sham, β-TCP, and Lenti-LacZ groups. Quantitative analysis (Fig. 4c) of the micro-CT data revealed that BMD was markedly higher in the Lenti-miRNA-21 group than in the sham, β-TCP, and Lenti-LacZ groups. In addition, BV/TV and bone mineral content (BMC) in the four groups followed similar patterns to that of BMD. These results indicate that miRNA-21 positively regulates BMSC ossification and that miRNA-21 constructs can markedly increase bone regeneration in canine mandibles.

### Histological analysis of miRNA-21-induced bone regeneration in canine mandibular defects

**Fig. 5:**
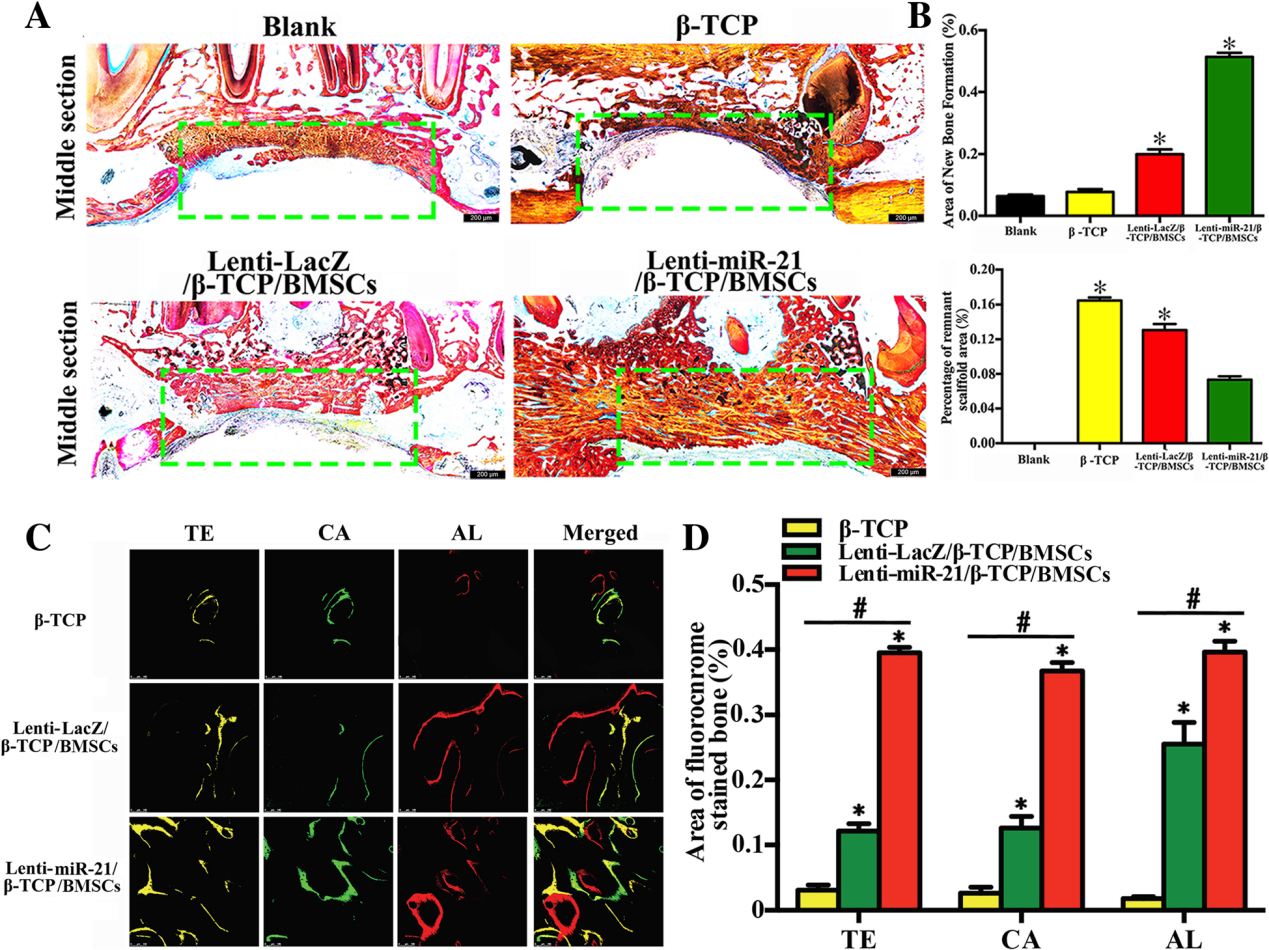
Histological analysis and fluorochrome labeling of miRNA-21-induced bone regeneration in canine mandibular defects. **a** Histological observations of undecalcified sections stained with van Gieson’s picrofuchsin from the four groups. **b** Histomorphometric measurements of the four groups (three animals in each group). The experiment was repeated three times. **c** Sequential fluorescence labeling of β-TCP, Lenti-LacZ/β-TCP/BMSC, and Lenti-miRNA-21/β-TCP/BMSC scaffolds. **d** Quantitative analysis of β-TCP, Lenti-LacZ/β-TCP/BMSC, and Lenti-miRNA-21/β-TCP/BMSC scaffolds. Yellow, green, and red represent labeling by tetracycline hydrochloride (TE), calcein (CA), and Alizarin Red (ARS), respectively (three animals in each group). The experiment was repeated three times. The bone area stained with TE, CA, and ARS was calculated (*p < 0.05; ^#^*p* < 0.05)

The newly formed bone in canine mandibular defects was examined with van Gieson’s staining. Substantial bone formation was observed in the Lenti-miRNA-21/β-TCP/BMSC group and less in the Lenti-LacZ/β-TCP/BMSC group. No obvious bone formation was found in the β-TCP or sham control groups (Fig. 5a). Sections from the Lenti-miRNA-21/β-TCP/BMSC group displayed a large amount of organized, mineralized bone tissue with lamellar morphology. In contrast, histological analyses of the other three groups revealed a small amount of irregularly arranged, woven bone tissue with large bone lacunae and fibrous connective tissue interspersed among the defect sites.

Furthermore, the percentages of newly formed bone and the degradation rates of the materials were also evaluated (Fig. 5b). Based on light microscopy, the percentages of new bone area after 6 months were 4.55 ± 1.12% in the sham group, 6.18 ± 1.22% in the β-TCP group, 21.36 ± 2.62% in the Lenti-LacZ group, and 52.21 ± 3.87% in the Lenti-miRNA-21 group, respectively. In addition, the percentages of the β-TCP residual area were 0 in the sham group, 16.86 ± 1.04% in the β-TCP group, 13.32 ± 2.48% in the Lenti-LacZ group, and 6.82 ± 1.43% in the Lenti-miRNA-21 group. Overall, these findings indicated that miRNA-21 can promote new bone formation in the canine mandibular defect area.

### Fluorochrome labeling and histomorphometric analysis

New bone formation and mineralization were assessed histomorphometrically via tetracycline, calcein, and ARS fluorescence, which represent mineralization fronts at specific time points (Fig. 5c, d). As shown in Fig. 5d, by week 2, the percentage of tetracycline labeling (yellow) in the Lenti-miRNA-21 group was 38.62 ± 2.26%, which was greater than that in the β-TCP or Lenti-LacZ group. After 4 months, the percentages of calcein labeling (green) were 4.46 ± 2.68%, 13.43 ± 3.22%, and 34.52 ± 2.88% for the β-TCP, Lenti-LacZ, and Lenti-miRNA-21 groups, respectively. The Lenti-miRNA-21 group was significantly different from the Lenti-LacZ and β-TCP groups, but no significant differences were identified between the β-TCP and Lenti-LacZ groups. After 6 months, the percentages of ARS labeling (red) were 2.23 ± 0.24%, 24.36 ± 4.42%, and 37.32 ± 3.18% for the β-TCP, Lenti-LacZ, and Lenti-miRNA-21 groups, respectively, and the labeling followed the same pattern as that of ALP staining. Taken together, these data indicate that Lenti-miRNA-21 enhanced ossification in the induced BMSCs and effectively promoted new bone formation.

## Discussion

In previous study, we have found that miRNA-21 can promote angiogenesis in human umbilical cord blood-derived mesenchymal stem cells (UCBMSCs) [14]. However, the role of miRNA-21 in osteogenesis was not clear. Therefore, the present study aimed to investigate the role of miRNA-21 in osteogenesis. Also, considering that BMSCs had higher proliferative ability and greater osteogenic differentiation potential compared with adipose tissue-derived stem cells compared (ADSCs) [28]. In this study, we chose the BMSCs as the tool and performed the experiments to investigate the role of miRNA-21 in the osteogenic differentiation of BMSCs.

In the present study, the role of miRNA-21 in osteogenic differentiation of bone marrow-derived stem cells (BMSCs) was investigated. In this study, the essential role of miRNA-21 in bone regeneration was explored in vitro and in vivo. It was found that miRNA-21 promotes BMSC migration and HIF-1α activity. miRNA-21 upregulated the expression level of P-Akt, an important marker of the PTEN/PI3K/Akt signaling pathway. Moreover, miRNA-21 overexpression increased BMSC-mediated osteogenesis and bone regeneration in both rat skulls and canine mandibular bone defects. These results indicate that miRNA-21 can promote bone regeneration via the PTEN/PI3K/Akt/HIF-1α pathway.

Initially discovered in 1993, miRNAs are small, noncoding RNA molecules (of approximately 17–25 nucleotides) found in plants, animals, and some viruses that silence RNA and posttranscriptionally regulate gene expression [7]. miRNAs play important roles in cell proliferation, differentiation, and apoptosis, particularly in cancer cells [29–31]. miRNA-21 has been previously reported to be highly expressed in many tumor cells, participating in carcinogenesis, tumor progression, and metastasis [32]. In our previous work, we found that miRNA-21 promoted angiogenesis in human UCBMSCs by enhancing HIF-1α activity [14]. Therefore, we hypothesized that miRNA-21 may also play an important role in bone regeneration by enhancing HIF-1α activity. In the present study, miRNA-21 markedly enhanced BMSC migration compared with that in the control group. Furthermore, miRNA-21-modified BMSCs showed improved osteoblast differentiation. We also detected the mRNA and protein expression levels of four key osteogenic factors, BMP-2, Runx2, OCN, and OPN, in gene-modified BMSCs, all of which were upregulated in the Lenti-miRNA-21 group. These results indicate that miRNA-21 promoted BMSC osteogenic differentiation in vitro.

A variety of target genes has been reported to be regulated by miRNA-21 [14, 33]. Among the target genes of miRNA-21, the PTEN/PI3K/Akt pathway—also known as the PI3K/Akt pathway—has been implicated as an important regulator of osteoblast differentiation [14]. However, no studies have investigated the relationship between miRNA-21 and the PTEN/PI3K/Akt pathway in bone regeneration. Interestingly, we found that miRNA-21 overexpression upregulated P-Akt expression and HIF-1α activity and inhibited PTEN. HIF-1α and P-Akt protein levels were also upregulated in miRNA-21-overexpressing BMSCs in vitro. However, when a PI3K inhibitor was added, P-Akt and HIF-1α protein expression levels in miRNA-21-overexpressing BMSCs were dramatically decreased. Therefore, we concluded that miRNA-21 overexpression promoted HIF-1α activity via the PTEN/PI3K/Akt pathway. Using an in vivo rat bone defect model, we showed that miRNA-21 overexpression increased the osteogenic ability of BMSCs. Micro-CT observations revealed that bone regeneration was enhanced in the miRNA-21-overexpressing group compared with that in the control group. Furthermore, fluorochrome labeling and histomorphometric analysis demonstrated that miRNA-21 significantly increased new bone formation and mineralization in bone defect sites. Histologically, an increased amount of organized, mineralized bone tissue with lamellar bone morphology was observed in the miRNA-21/β-TCP/BMSC scaffold group. Thus, the present study has uncovered that miRNA-21 enhances BMSC osteogenesis via increasing HIF-1α and P-Akt activity, and PTEN degradation, resulting in upregulated new bone formation in rat and canine bone defect sites.

## Conclusion

Our work demonstrated an essential role for miRNA-21 in promoting bone regeneration. miRNA-21 overexpression increased BMSC migration and HIF-1α activity via the PTEN/PI3K/Akt pathway, which increased BMSC osteogenic differentiation to promote bone regeneration in bone defects. miRNA-21 overexpression in BMSCs may offer great therapeutic promise for maxillofacial bone defect reconstruction.

## Additional file

Additional file 1: Additional "Materials and Methods" and "Results" of this study. (DOCX 1439 kb)

## Abbreviations

Akt: RAC-beta serine/threonine-protein kinase
ALP: Alkaline phosphatase
ARS: Alizarin Red S
BMDs: Bone mineral densities
BMP-2: Bone morphogenetic protein 2
BMSCs: Bone marrow-derived stem cells
CSD: Critical size defects
EDTA: Ethylenediaminetetraacetic acid
FBS: Fetal bovine serum
HIF-1α: Hypoxia-inducible factor-1α
miRNA-21: MicroRNA-21
OCN: Osteocalcin
OPN: Osteopontin
PI3K: Phosphoinositide-3-kinase
PTEN: Phosphatase and tensin homolog
qPCR: Quantitative reverse-transcription polymerase chain reaction
Runx2: Runt-related transcription 2
SD: Standard deviation
TbN: Trabecular number
TbTh: Trabecular thickness
UCBMSCs: Umbilical cord blood-derived mesenchymal stem cells
VEGF: Vascular endothelial growth factor
β-TCP: β-Tricalcium phosphate
