# Supplementary material for: miRNA-21 promotes osteogenesis via the PTEN/PI3K/Akt/HIF-1α pathway and enhances bone regeneration in critical size defects
Source: Stem Cell Res Ther. 2019 Feb 22;10:65. doi: 10.1186/s13287-019-1168-2 (PMC6387542; doi:10.1186/s13287-019-1168-2)
Supplement: Supplementary file 1 — Additional "Materials and Methods" and "Results" of this study. (DOCX 1439 kb) [file 13287_2019_1168_MOESM1_ESM.docx]

**Role of miRNA-21 Modified Bone Marrow-Derived Stem Cells in Promoting Osteogenesis via PTEN-PI3K/Akt/HIF-1α Pathway and Enhancing Bone Regeneration in Critical-Size Defects**

**Appendix**

**Materials and Methods**

*In vitro migration assays*

BMSCs were seeded and grown in 24-well plates (3 × 10^4^ cells/well) for target gene transfection. Consistent, 400- to 500-µm wounds were generated in confluent cell layers using a pipette tip. Cell nuclei were stained with Hoechst (Life Technologies, USA) and then cells were observed via microscopy. After 24 and 48 h, migration was monitored in real-time using a microscope.

**Real-time PCR assays**

miRNA-21 primers used were as follows: forward, caaagatcactatcccaatcatc and reverse, gcggtctttctcaatctaagtc.

qPCR primer sequences used were as follows:

HIF-1α, 5ʹ-gtgacatgatttacatttctg-3ʹ and 5ʹ-tcacaaggccatttctgtgtg-3ʹ;

VEGF, 5ʹ-atgtctatcagcgcagctact-3ʹ and 5ʹ-tccgcataatctgcatggtga-3ʹ;

BMP-2, 5ʹ-accatggattcgtggtggaag-3ʹ and 5ʹ-gacttaaggcgtttccgctgt-3ʹ;

Osteocalcin, 5ʹ-tttgcatcgctggccaggcag-3ʹ and 5ʹ-ccggattgagctcacacacct-3ʹ;

Runx2, 5ʹ-actcactaccacacctacctg-3ʹ and 5ʹ-aatagcgtgctgccattcgag-3ʹ;

OPN, 5ʹ-aagccatgaccacatggatga-3ʹ and 5ʹ-aacttcggttgctggcaggtc-3ʹ;

GAPDH, 5ʹ-ggtcggagtcaacggatttgg-3ʹ and 5ʹ-cttccaggagcgagatccctc-3ʹ.

**Alkaline phosphatase (ALP) activity and calcium deposition assays**

BMSCs, Lenti-LacZ/BMSCs, and Lenti-miRNA-21/BMSCs were seeded in DMEM in 24-well plates at a density of 5.0 × 10^4^ cells/mL. After 14 d of incubation, each group of cells was fixed and stained using an ALP kit (Beyotime, China) or Alizarin Red S (ARS). The cells were washed five times with PBS and observed under an optical microscope.

**Results**

**miRNA-21 increased BMSC migration**

A scratch wound assay was performed using BMSCs transfected with control or miRNA-21 constructs to determine the role of miRNA-21 in BMSC migration. The results revealed that BMSCs with miRNA-21 overexpression possessed increased migratory capabilities (Figure S1A), while migratory capabilities of BMSCs treated with a miRNA-21 inhibitor were markedly reduced compared with those of the control BMSCs (Figure S1B).


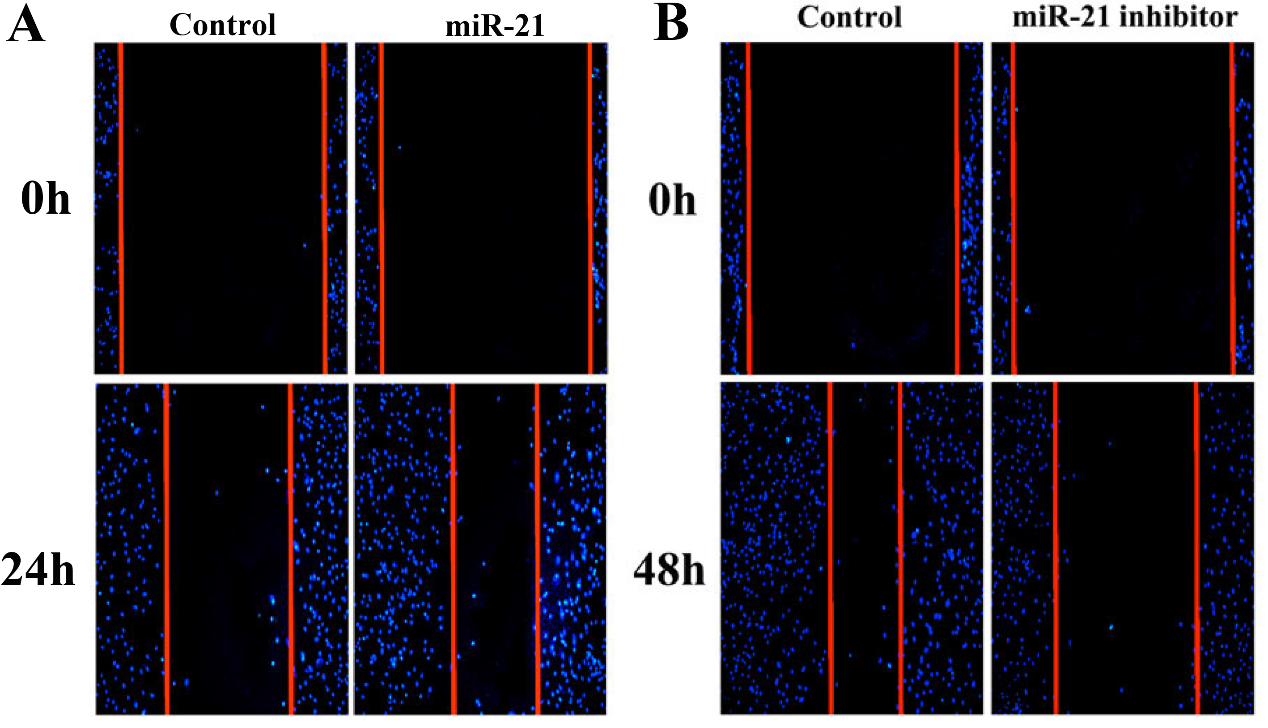


**Figure S1** The role of miRNA-21 in directed migration of BMSCs. (A) Migration of BMSCs transfected with a control or miRNA-21 mimic. (B) Migration of BMSCs transfected with a control or miRNA-21 inhibitor.

**Increased ALP expression and calcium deposition in miRNA-21-transduced BMSCs**

To study how miRNA-21 promotes osteogenesis, staining methods were used to investigate miRNA-21 regulation of ALP expression and calcium deposition in BMSCs. BMSCs, Lenti-miRNA-21-transduced BMSCs, and LacZ-transduced BMSCs were plated on 6-well plates (1 × 10^5^ cells/well). At 21 d after gene transduction, ALP staining was performed. ALP expression significantly increased in miRNA-21-transduced groups (Figure S2). Similarly, ARS staining revealed a significant increase in calcium deposition on day 21 (Figure S2). These results confirmed that miRNA-21 promoted BMSC osteoblast differentiation.


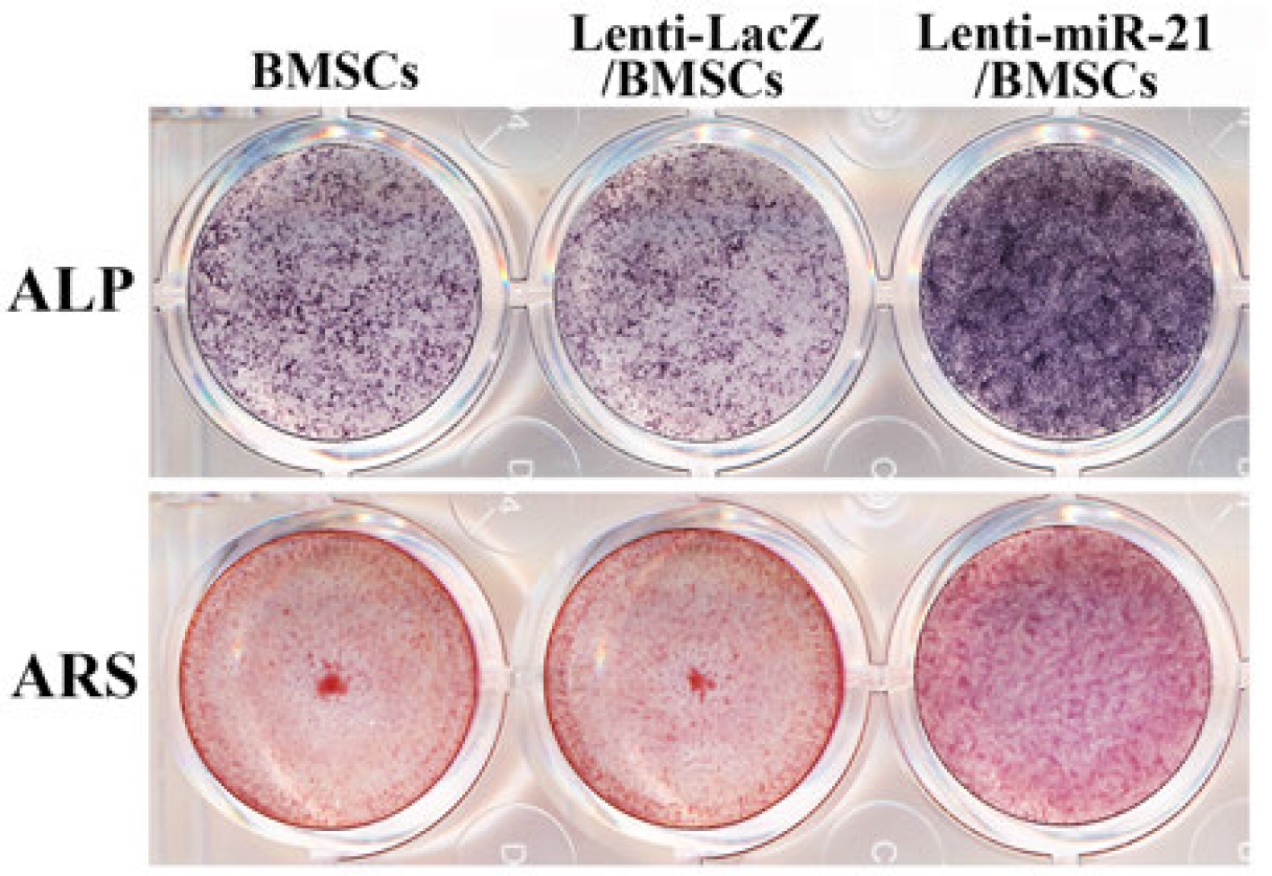


**Figure S2** ALP and Alizarin Red S staining were determined in BMSCs transfected with Lenti-LacZ/BMSCs or Lenti-miRNA-21/BMSCs.

**Surgical procedure**

Surgical procedure of the canine mandibular defect model was shown in Figure S3.


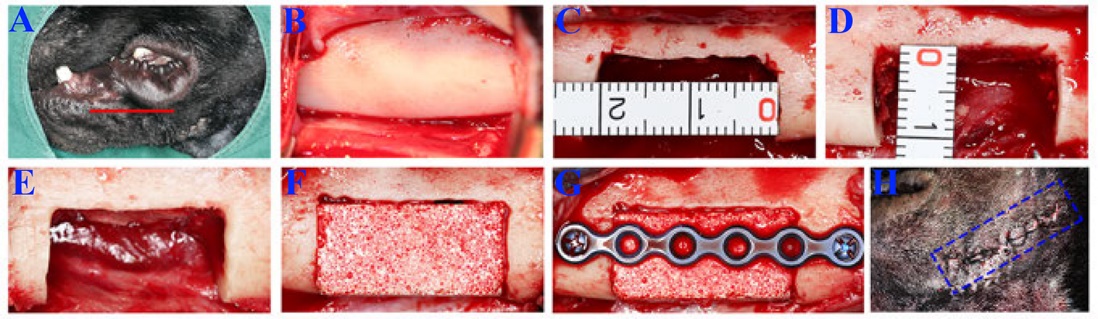


**Figure S3** Animal surgery for the canine mandibular defect model. (A) Operative incision. (B–E) Creation of the canine mandibular defect model. (F, G) Placement of constructs in the defect area. (H) Closure of the incisions.
